# Influenza a virus antagonizes type I and type II interferon responses via SOCS1-dependent ubiquitination and degradation of JAK1

**DOI:** 10.1186/s12985-020-01348-4

**Published:** 2020-06-12

**Authors:** Yinping Du, Fan Yang, Qiuxia Wang, Nuo Xu, Yizhang Xie, Sujuan Chen, Tao Qin, Daxin Peng

**Affiliations:** 1 College of Veterinary Medicine, Yangzhou University, Yangzhou, Jiangsu 225009 People’s Republic of China; 2 Jiangsu Co-Innovation Center for the Prevention and Control of Important Animal Infectious Disease and Zoonoses, Yangzhou, Jiangsu 225009 People’s Republic of China; 3 Jiangsu Research Center of Engineering and Technology for Prevention and Control of Poultry Disease, Yangzhou, Jiangsu 225009 People’s Republic of China

**Keywords:** IAV, JAK1, IFN, SOCS1, Degradation

## Abstract

**Background:**

Although influenza A virus (IAV) employs diverse strategies to evade IFN responses by inhibiting the synthesis of IFN, how IAV regulates signaling downstream of IFN is incompletely understood.

**Methods:**

In this study, we used Western blot-based protein analysis coupled with RT-qPCR, overexpression and RNA interference to investigate the regulation of JAK1 by IAV infection.

**Results:**

The results indicated that JAK1 was ubiquitinated and degraded, resulting in inhibition of type I and type II IFN responses, demonstrating that IAV antagonizes the IFN-activated JAK/STAT signaling pathway by inducing the degradation of JAK1. Furthermore. IAV infection upregulated the suppressor of cytokine signaling (SOCS) protein SOCS1, and SOCS1 mediated the ubiquitination and degradation of JAK1.

**Conclusion:**

Collectively, our findings suggest that IAV infection induces SOCS1 expression to promote JAK1 degradation, which in turn inhibits host innate immune responses.

## Background

The emergence of influenza A virus causing significant morbidity and mortality in people remains a global health concern. Influenza A virus naturally circulate in the wild bird population, such as waterfowl and ducks, and can spill over to other species, including humans [1]. Outbreaks of avian influenza virus such as H5N1, H7N9, and H9N2 virus have caused high morbidity and mortality rates in humans, raising the risk for the occurrence of influenza pandemics [2–4]. Antiviral drugs are available for treating influenza, but numerous strains of IAV are resistant, presumably due to mutation. Thus, identifying mechanisms for IAV regulation of host immunity and designing new therapeutic strategies are important to effectively control influenza.

Influenza virus infection can be sensed by host cellular pathogen recognition receptors (PRRs), which in turn activate downstream signaling cascades and then induce the expression of cytokines, including interferons (IFNs) [5]. IFNs are a superfamily of cytokines which are classified into type I, type II, and type III subtypes. IFNs and interferon-stimulated genes (ISGs) establish a crucial line of antiviral defense, inhibiting virus replication and restricting the spread of viruses [6]. After being secreted, the IFNs bind to the cognate IFN receptors to initiate the JAK/STAT signaling pathway, involving tyrosine kinases of JAK family and transcription factors of STAT family [6, 7]. Activation of JAK/STAT pathway leads to the induction of various ISGs, and some ISGs have direct anti-influenza virus activities [8]. Previous studies using IFN receptors or STAT1 gene knockout mice have demonstrated the importance of IFNs response to anti-influenza defense [9–11].

It is not well understood how IAV regulate the IFN induced JAK/STAT signaling pathway. It was reported that IAV downregulated IFN receptors level upon infection, and then inhibited the antiviral activity of IFNs [12]. IAV infection induced SOCS1 could inhibit the activity of STAT1 [13]. However, it is unknown whether and how IAV regulates the JAK1 protein downstream of IFN receptors. Some viruses induced the degradation of JAK1, and then inhibited the IFNs stimulated antiviral and immunoregulatory activity [14–17]. In this study, we investigated whether IAV infection regulated JAK1.

We found that IAV infection significantly downregulated the protein level of JAK1. IAV infection facilitated the ubiquitination of JAK1 to promote its degradation. Rescued JAK1 expression could restore the IFNs induced phosphorylation of STAT1 and the expression of ISGs. Those results indicated that IAV facilitated its replication by inducing the degradation of JAK1 during infection. We further showed that IAV infection upregulated SOCS1 expression, and SOCS1 mediated JAK1 ubiquitination and proteasome dependent degradation. These data extend our knowledge of influenza pathogenesis and suggest new therapeutic targets for treating influenza.

## Materials and methods

### Virus and cells

Three Influenza A virus isolates A/mallard/Huadong/S/2005 (H5N1) [18], A/chicken/Jiangsu/WJ-14/2015 (H7N9) [19] and A/chicken/Taixing/10/2010 (H9N2) [20] were used in this study. Viruses were amplified in 10-day-old specific-pathogen-free (SPF) chicken embryonated eggs. Virus yields were quantified using TCID50 assays on MDCK cells. After adsorption at 37 °C for 1 h in 5% CO_2_, the virus-infected MDCK cells were maintained in minimum Eagle’s medium (MEM; Gibco) containing 1% FBS (Gibco) and 0.5 μg/ml tosylphenylalanyl chloromethyl ketone (TPCK)-treated trypsin (Sigma-Aldrich). Human lung epithelial A549 cells, human embryonic kidney 293 T cells, and MDCK cells were cultured in Dulbecco’s modified Eagle’s medium (DMEM; Gibco) with 10% FBS (Gibco) and penicillin (100 U/ml)–streptomycin (100 μg/ml) (Invitrogen).

### Reagents and antibodies

Cycloheximide (CHX; Sigma-Aldrich), anti-DYKDDDDK (Flag) G1 Affinity Resin (GenScript), phenylmethylsulfonyl fluoride (PMSF) (Gold Bio), immunoprecipitation (IP) lysis buffer (Thermo Scientific), TPCK-treated trypsin (Sigma-Aldrich), proteasome inhibitor MG132 (Carbobenzoxy-L-leucyl-L-leucyl-L-leucinal, Selleck chem), NH_4_Cl (Ammonium chloride, Selleck chem), and recombinant human IFN-α2 (GenScript) and IFN-γ (GenScript) were purchased from the indicated manufacturers. Antibodies against JAK1, STAT1, phospho-STAT1, and β-actin were purchased from Sigma-Aldrich; antibodies against SOCS1 and SOCS3 were purchased from GeneTex, antibodies against influenza virus NP, M1, and NS1 were purchased from GeneTex; antibodies against DYKDDDDK (Flag) tag and HA tag were purchased from Cell Signaling Technology. Human SOCS1 siRNAs si-1, GCAUCCGCGUGCACUUUCAdTdT, and si-2, CUACCUGAGCUCCUUCCCCdTdT were synthesized by Gene Pharma.

### Virus infection

A549 cells, 293 T cells and MDCK cells seeded in 1-ml volumes of medium at a density of 1 × 10^6^ cells/ml in 12-well plates were incubated with indicated IAV (A/mallard/Huadong/S/2005 (H5N1), A/chicken/Jiangsu/WJ-14/2015 (H7N9) and A/chicken/Taixing/10/2010 (H9N2)) at an MOI (multiplicity of infection) of 1 for 1 h, and then the virus were removed and the cells were cultured for 24 h. The low path (H9N2) infection was performed in the presence of TPCK-treated trypsin (1 μg/ml). Influenza A/mallard/Huadong/S/2005 (H5N1) was used for cellular adsorption in the majority of the following experiments at the indicated MOI for 1 h, and then the virus were removed and the cells were cultured for the indicated time before next step treatment. The experiments were independently repeated at least twice.

### Constructs and transfection

The human JAK1 gene was amplified using reverse transcription PCR (RT-PCR), and pcDNA3.1(+)-His and p3xFlag CMV-14 were used to construct plasmids encoding JAK1. Ub (ubiquitin) gene was amplified using reverse transcription PCR (RT-PCR) and cloned into the vector pCDNA3.1(+)-HA to construct HA-Ub. For transient expression in 293 T cells, cells were transfected with plasmids using PolyJet In Vitro DNA Transfection Reagent (Signa gene) at a ratio of PolyJet to DNA of 3:1, according to the protocol recommended by the manufacturer. Then the 293 T cells were incubated with the transfection complex at 37 °C for indicated times. For siRNA transfection, 293 T cells plated onto a 6-well plate were transfected with siRNAs targeting SOCS1 at a concentration of 100 pmol/ml using Lipofectamine 2000 (Thermo Fisher), according to the protocol recommended by the manufacturer. All the data presented were repeated at least twice in independent experiments.

### Western blot analysis

Cells were lysed in the 2x sample buffer (Beyotime) and heated at 100 °C for 10 min. Equal amounts of protein samples were resolved on a 10% sodium dodecyl sulfate–polyacrylamide gel electrophoresis (SDS-PAGE) gel and transferred to nitrocellulose membrane (Bio-Rad). Membrane-bound proteins were incubated with specific antibodies and detected using an enhanced chemiluminescence substrate (Thermo Scientific). All the data presented were repeated at least twice in independent experiments.

### Real-time PCR

Total cellular RNA was purified using Trizol Reagent (Sigma-Aldrich) according to the manufacturer’s instructions and was treated with DNase I (Thermo Scientific) to remove contaminated DNAs. The RNA was reverse transcribed using oligo-18dT (Invitrogen), and the resulting cDNA was then analyzed by real-time quantitative PCR (qPCR) using gene-specific primers. Primers for human JAK1 (5′-CTT TGC CCT GTA TGA CGA GAA C-3′ and 5′-ACC TCA TCC GGT AGT GGA GC-3′), Mx1 (5′-GTT TCC GAA GTG GAC ATC GCA-3′ and 5′-CTG CAC AGG TTG TTC TCA GC-3′), ISG56 (5′-AGA AGC AGG CAA TCA CAG AAA A-3′ and 5′-CTG AAA CCG ACC ATA GTG GAA AT-3′), TAP-1 (5′-TGT GAC AAG GTT CCC ACT GCT TAC3’ and 5′-GGC TGT GGC CTA TGC AGT CA-3′), LMP-2 (5′-GCA TAT AAG CCA GGC ATG TCT CC-3′ and 5′-AGC TGT AAT AGT GAC CAG GTA GAT GAC-3′), SOCS1 (5′-GAC ACT CAC TTC CGC ACC T-3’and 5′-GAA GAA GCA GTT CCG TTG G-3′), SOCS3 (5′-GCA GGA GAG CGG ATT CTA CT-3′ and 5′-ACG CTC AAC GTG AAG AAG TG-3′)were used. QPCRs were performed with SYBR green I chemistry using a Step One Plus Real-Time PCR instrument. The comparative Ct [2(−ΔΔCt)] method was used to analyze gene expression, and genes quantities were normalized to the housekeeping gene (GAPDH, 5′-TCATCATCTCTGCCCCTTCT-3′ and 5′-GTCATGAGTCCCTCCACGAT-3′). All the data presented were repeated at least twice in independent experiments.

### Immunoprecipitation and ubiquitination assay

For detection of ubiquitination of JAK1 during IAV infection, 293 T cells were transfected with Flag-JAK1 (1 μg) and tHA-Ub (0.5 μg). At 24 h post transfection, cells were left uninfected or infected with IAV at an MOI of 1 for an additional 18 h. For IP experiments, these cells were lysed with IP lysis buffer containing protease inhibitor (PMSF, 1 mM) and incubated with 20 μl Anti-DYKDDDDK G1 affinity resin overnight under rotation at 4 °C. The beads were washed three times with IP lysis buffer. The beads were washed, and the precipitates were analyzed by Western blotting. The experiments were independently repeated twice with similar results.

### Statistical analysis

Prism version 5.0 (GraphPad) was used for data analysis. Western blotting data was quantified by the software, Image-Pro Plus 6.0. Statistical significance of real-time quantitative PCR data were analyzed using unpaired Student’s t-test for continuousvariables. A p value less than 0.05 was considered to be significant.

## Results

### Influenza a virus infection down regulates JAK1

**Fig. 1:**
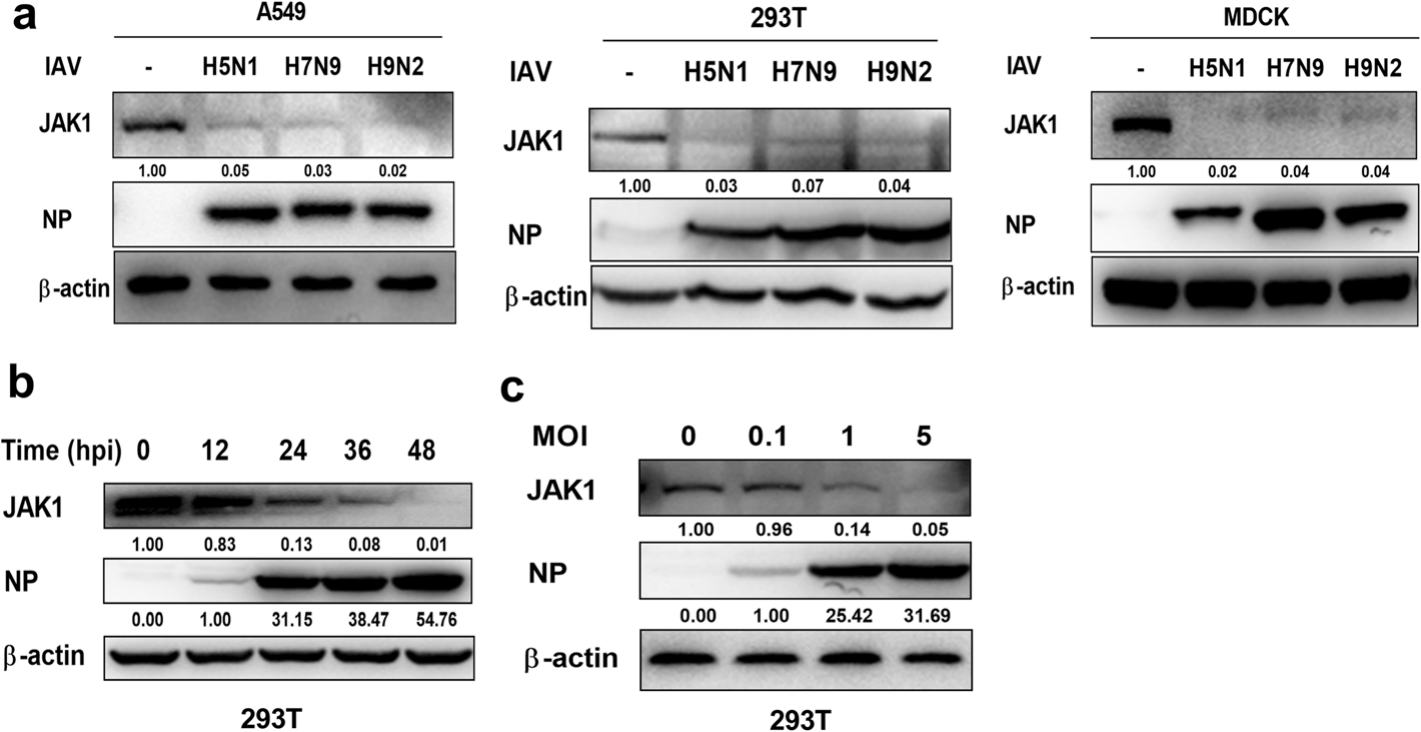
IAV induces the degradation of JAK1. (**a**) A549 cells, 293 T cells and MDCK cells were infected or uninfected (Mock) with indicated IAV (A/mallard/Huadong/S/2005 (H5N1), A/chicken/Jiangsu/WJ-14/2015 (H7N9) and A/chicken/Taixing/10/2010 (H9N2)) at an MOI of 1 for 24 h, The protein levels of JAK1 and viral NP were analyzed by Western blotting. The protein level of β-actin was used as internal loading control. (**b**) 293 T cells were left uninfected or infected with IAV influenza A/mallard/Huadong/S/2005 (H5N1), which was used for the majority of the following experiments unless indicated otherwise, at an MOI of 1 for the indicated times. The levels of JAK1, viral NP and β-actin were analyzed by Western blotting. (**c**) 293 T cells were left uninfected or infected with IAV at the indicated MOI for 24 h. The protein levels of JAK1, viral NP and β-actin were analyzed using Western blotting

To investigate the regulation of JAK1 during influenza A virus infection, JAK1 protein abundance was quantified using Western blotting. We infected cells with three different strain of influenza viruses: A/mallard/Huadong/S/2005 (H5N1), A/chicken/Jiangsu/WJ-14/2015 (H7N9) and A/chicken/Taixing/10/2010 (H9N2). Following all subtypes of IAV infection, the protein level of JAK1 was strongly decreased in both A549 cells, 293 T cells and MDCK cells (Fig. 1a). IAV infection-induced JAK1 downregulation was time- and virus dose-dependent, and the protein level of JAK1 was decreased during the replication of IAV, as assayed by the expression of viral NP in infected cells (Fig. 1b and c). These data demonstrate that IAV infection significantly downregulates the protein level of JAK1.

### IAV infection induces the ubiquitination and proteasome degradation of JAK1

**Fig. 2:**
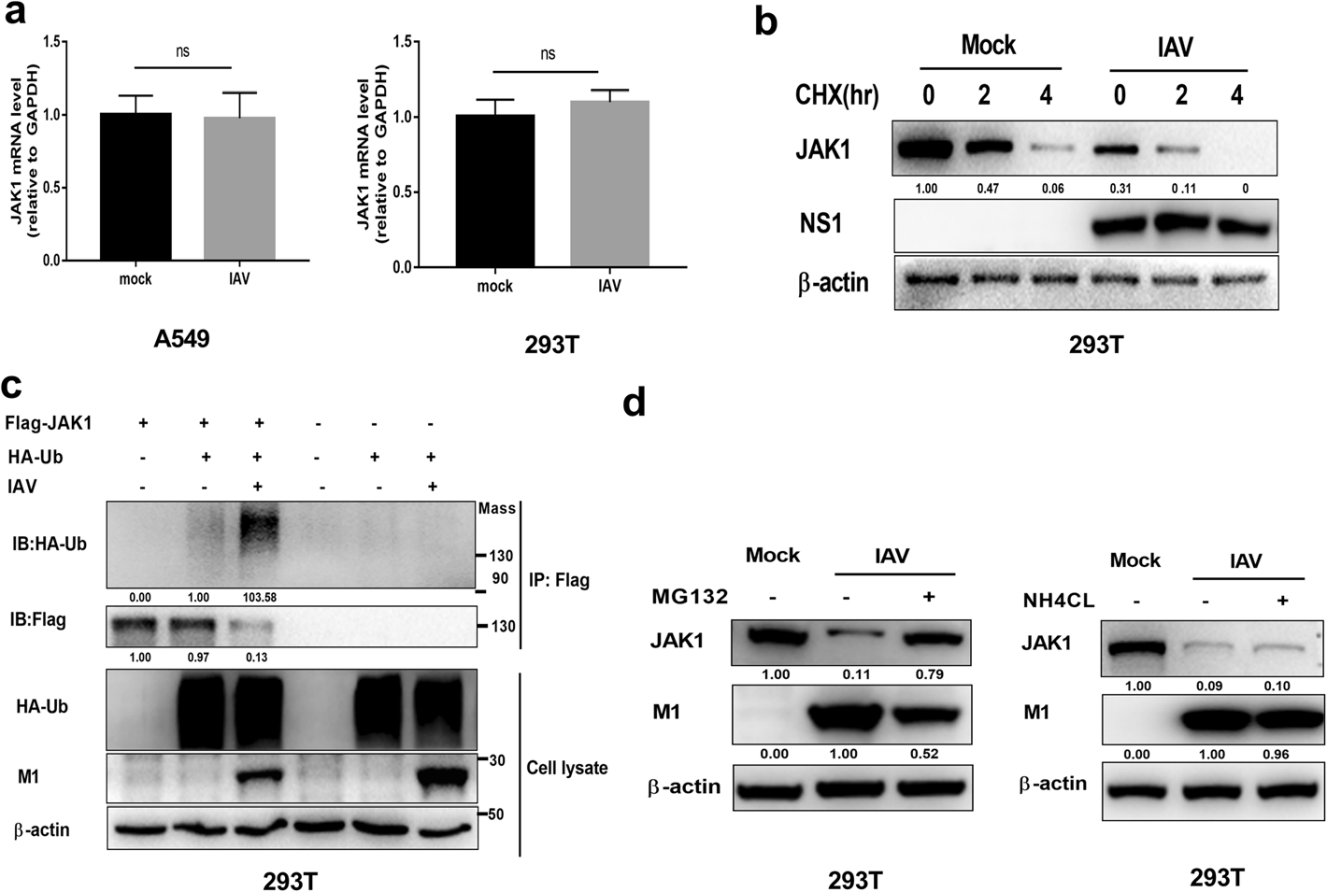
IAV infection induces ubiquitination and proteasome-mediated degradation of JAK1. (**a**) 293 T cells and A549 cells were left uninfected or infected with IAV at an MOI of 1. The relative mRNA levels of JAK1 were analyzed by real-time qPCR at 24 hpi. Date are means plus standard deviation for triplicate samples. The experiments were independently repeated twice with similar result. Ns, not significant. (**b**) 293 T cells were uninfected or infected with IAV at an MOI of 1. At 12 hpi, the cells were treated with solvent or cycloheximide (CHX, 50 μg/ml) for the indicated times. The levels of JAK1, viral NS1 and β-actin were analyzed using Western blotting. (c) 293 T cells were transfected with Flag-tagged JAK1 and HA-tagged ubiquitin, 24 h post transfection, cells were left uninfected or infected with IAV at an MOI of 1 as indicated for additional 18 h. Cell lysis was subjected to IP and Western blotting. The ubiquitination of immunoprecipitated Flag-JAK1 was analyzed by Western blotting using anti-HA tag antibody. The protein levels of Flag-JAK1, HA-Ub, β-actin, and viral M1 in the whole-cell lysates were also analyzed using Western blotting. (**d**) 293 T cells were infected with IAV at an MOI of 1. At 18 h post infection, cells were treated with indicated inhibitors or solvent (dimethyl sulfoxide [DMSO]) for an additional 6 h. The protein levels of JAK1, viral M1, and β-actin were analyzed using Western blotting

We determined the stage of JAK1 downregulation during IAV infection. First, the mRNA level of JAK1 in IAV infected cells and control cells were detected using real-time quantitative PCR. IAV infection had no effect on the mRNA level of JAK1 (Fig. 2a), suggesting that the inhibition of JAK1 expression occurred at the post-transcriptional stage. Then we treated IAV infected cells and control cells with the translation inhibitor cycloheximide (CHX) and then detected the protein stability of JAK1. The result showed that IAV infection accelerated JAK1 protein degradation (Fig. 2b). As the ubiquitin proteasome system was reported to mediate the degradation of JAK1 [21], we determined if IAV infection induces JAK1 ubiquitination. To detect the ubiquitination of JAK1, Flag-tagged JAK1 and HA-tagged Ub were transiently overexpressed in 293 T cells, and the ubiquitination of JAK1 was assessed using immunoprecipitation and Western blotting. IAV infection increased the ubiquitination level of JAK1, indicating that JAK1 ubiquitination is induced by IAV infection (Fig. 2c). Proteasome-dependent and lysosome-dependent protein degradation pathway are two distinct protein degradation mechanisms. We sought to investigate the JAK1 degradation pathway induced by IAV infection. Following infection with IAV, cells were treated with MG-132 (proteasome inhibitor) or NH_4_Cl (lysosome inhibitor). The degradation of JAK1 induced by IAV was repressed by MG-132 but not by NH_4_Cl, suggesting that proteasome-dependent pathway mediated the degradation of JAK1 during IAV infection (Fig. 2d).

### IAV infection induces cell less responsive to type I and type II IFNs

**Fig. 3:**
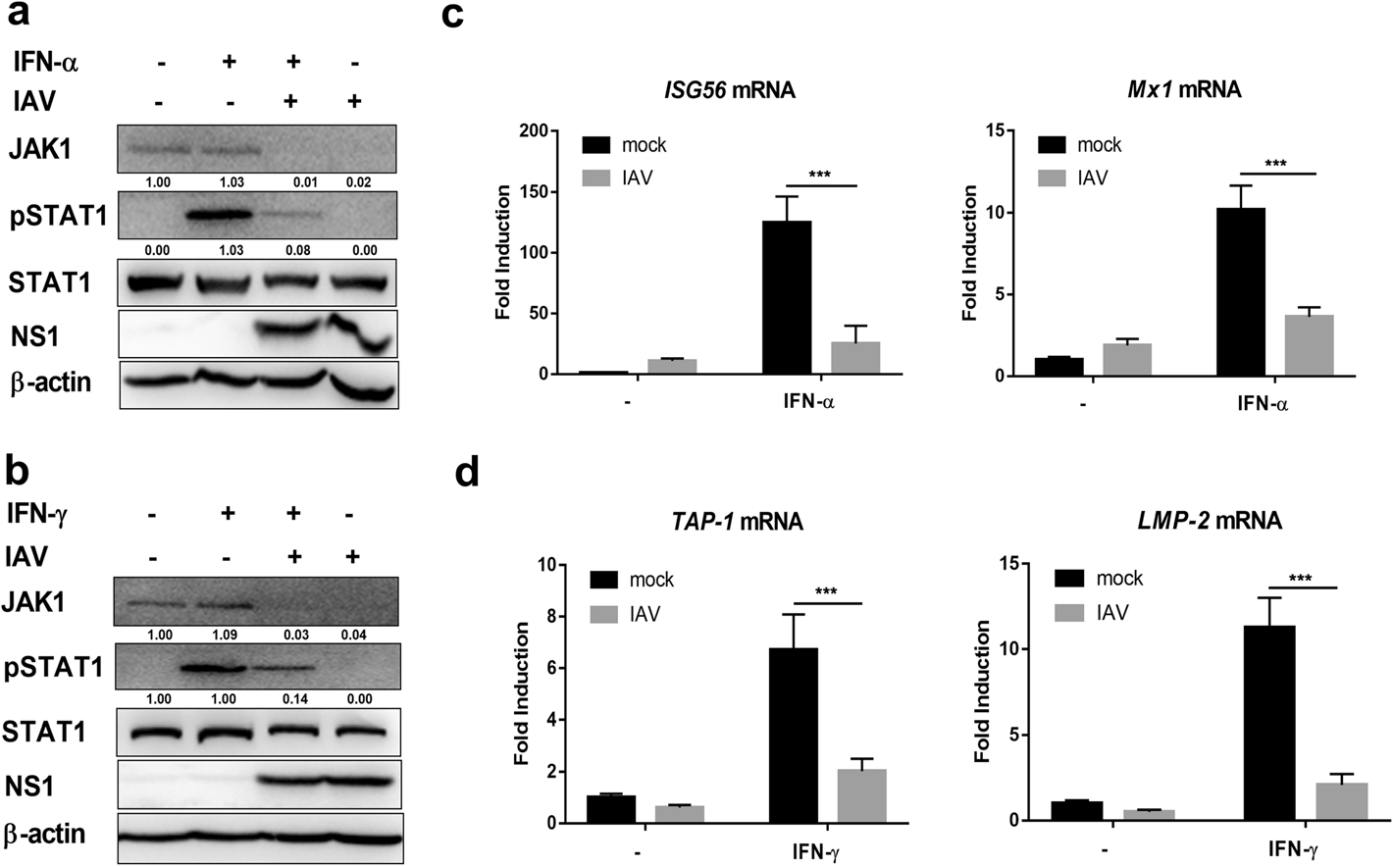
IAV infection reduces responsiveness to IFNs. (**a**) 293 T cells were left uninfected (Mock) or infected with IAV at an MOI of 1. 24 h post infection, cells were left untreated (−) or treated with human IFN-α (1000 U/ml) for 1 h. The levels of JAK1, pSTAT1, STAT1, viral NS1, and β-actin were detected using Western blotting. (**b**) 293 T cells were left uninfected (Mock) or infected with IAV at an MOI of 1. At 24 hpi, cells were left untreated (−) or treated with human IFN-γ (1000 U/ml) for 1 h. The levels of JAK1, pSTAT1, STAT1, viral NS1, and β-actin were detected using Western blotting. (**c**) 293 T cells were left uninfected (Mock) or infected with IAV at an MOI of 1. At 24 hpi, cells were left untreated (−) or treated with human IFN-α (1000 U/ml) for 24 h. The relative mRNA levels of Mx1 and ISG56 were analyzed using real-time qPCR. The error bars represent the means plus standard deviations for three independent experiments. ***, P < .0001. (d) 293 T cells were left uninfected (Mock) or infected with IAV at an MOI of 1. At 24 hpi, cells were left untreated (−) or treated with IFN-γ (1000 U/ml) for 6 h. The relative mRNA levels of TAP-1 and LMP-2 were analyzed using real-time qPCR. The error bars represent the means plus standard deviations for three independent experiments. ***, *P* < .0001

Jak1 and the downstream transcription factors are required for the cellular responses to IFNs. Given the ability of IAV to reduce the protein level of JAK1, we sought to gain insight into the influence of IAV in IFNs mediated signaling pathway. We first investigated the activation of transcription factor STAT1 in response to type I and type II IFNs in the presence or absence of IAV infection. Treatment with recombinant human IFN-α or IFN-γ induced STAT1 activation, eliciting high levels of pSTAT1 (Fig. 3a and b). However, IAV infection strongly inhibited STAT1 phosphorylation after stimulation with IFN-α or IFN-γ, and at the same time the protein level of JAK1 was significantly downregulated (Fig. 3a and b). IFNs treatment can induce the expression of antiviral ISGs through JAK/STAT pathway, and ISGs play key rule in and immune regulation and controlling virus replication [6, 22]. We then determined whether IAV infection induced JAK1 degradation regulates the expression of ISGs. The transcription profiling of ISGs in response to IFN-α and IFN-γ was analyzed using qPCR. IAV infection substantially inhibited IFN-α induced expression of ISG56 and Mx1, as well as IFN-γ triggered Tap-1 and Lmp-2 (Fig. 3c and d). Collectively, these results indicate that IAV infection induces JAK1 degradation and decreases cellular sensitivity to IFNs.

### Rescuing JAK1 expression inhibits IAV replication

**Fig. 4:**
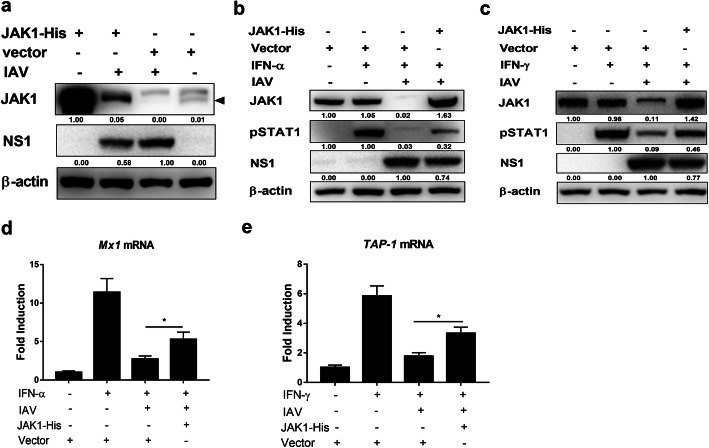
Rescuing JAK1 overexpression enhances cellular responses to type I and type II IFNs. (**a**) 293 T cells were transfected with control vector or plasmid encoding JAK1-His. At 24 h post-transfection, cells were infected with IAV at an MOI of 1 for an additional 24 h. The level of JAK1, viral NS1, and β-actin were detected using Western blotting. (**b**) 293 T cells were transfected with control vector or plasmid encoding JAK1-His. At 24 h post transfection, cells were infected with IAV at an MOI of 1. At 24 hpi, cells were left untreated (−) or treated with human IFN-α (1000 U/ml) for 1 h. The level of JAK1, pSTAT1, viral NS1, and β-actin were detected using Western blotting. (**c**) 293 T cells were transfected with control vector or plasmid encoding JAK1-His. At 24 h post transfection, cells were infected with IAV at an MOI of 1. At 24 hpi, cells were left untreated (−) or treated with human IFN-γ (1000 U/ml) for 1 h The level of JAK1, pSTAT1, viral NS1, and β-actin were detected using Western blotting. (d) 293 T cells were transfected with control vector or plasmid encoding JAK1-His. At 24 h post transfection, cells were left uninfected (Mock) or infected with IAV at an MOI of 1. At 24 hpi, cells were left untreated (−) or treat with human IFN-α (1000 U/ml) for 24 h. The relative mRNA levels of Mx1 were analyzed using real-time qPCR. The error bars represent the means plus standard deviations for three independent experiments. *, p ≤ 0.05. (e) 293 T cells were transfected with control vector or plasmid encoding JAK1-His. At 24 h post transfection, cells were left uninfected (Mock) or infected with IAV at an MOI of 1. At 24 hpi, cells were left untreated (−) or treat with human IFN-γ (1000 U/ml) for 6 h. The relative mRNA levels of TAP-1 were analyzed using real-time qPCR. The error bars represent the means plus standard deviations for three independent experiments. *, p ≤ 0.05

To further demonstrate the association of JAK1 degradation and IFNs response attenuation during IAV infection, 293 T cells were transfected with JAK1-His or control vector. Most of the overexpressed JAK1 was degraded during IAV infection, and JAK1 overexpression downregulated the expression of IAV protein NS1(Fig. 4a) and NP (data not shown), indicating that JAK1 degradation induced by IAV infection was beneficial to virus replication. JAK1 overexpression partially restored the activation of STAT1 in response to IFN-α and IFN-γ in the presence of IAV infection (Fig. 4b and c). The mRNA level of ISGs were also increased by JAK1 overexpression (Fig. 4d and e). Transient expression of JAK1 cannot fully restore the phosphorylation of STAT1 as well as the expression of ISGs. That may be because IAV infection can also inhibit the IFNs response by other ways, such as by decreasing the protein level of IFN receptors in several cell types [12]. Collectively, overexpression of JAK1 can partly restore cellular response to type I and type II IFNs and display anti influenza activity, suggesting that JAK1 degradation play a critical role in attenuating the antiviral activity of IFNs during IAV infection.

### IAV infection induced SOCS1 mediate the degradation of JAK1

**Fig. 5:**
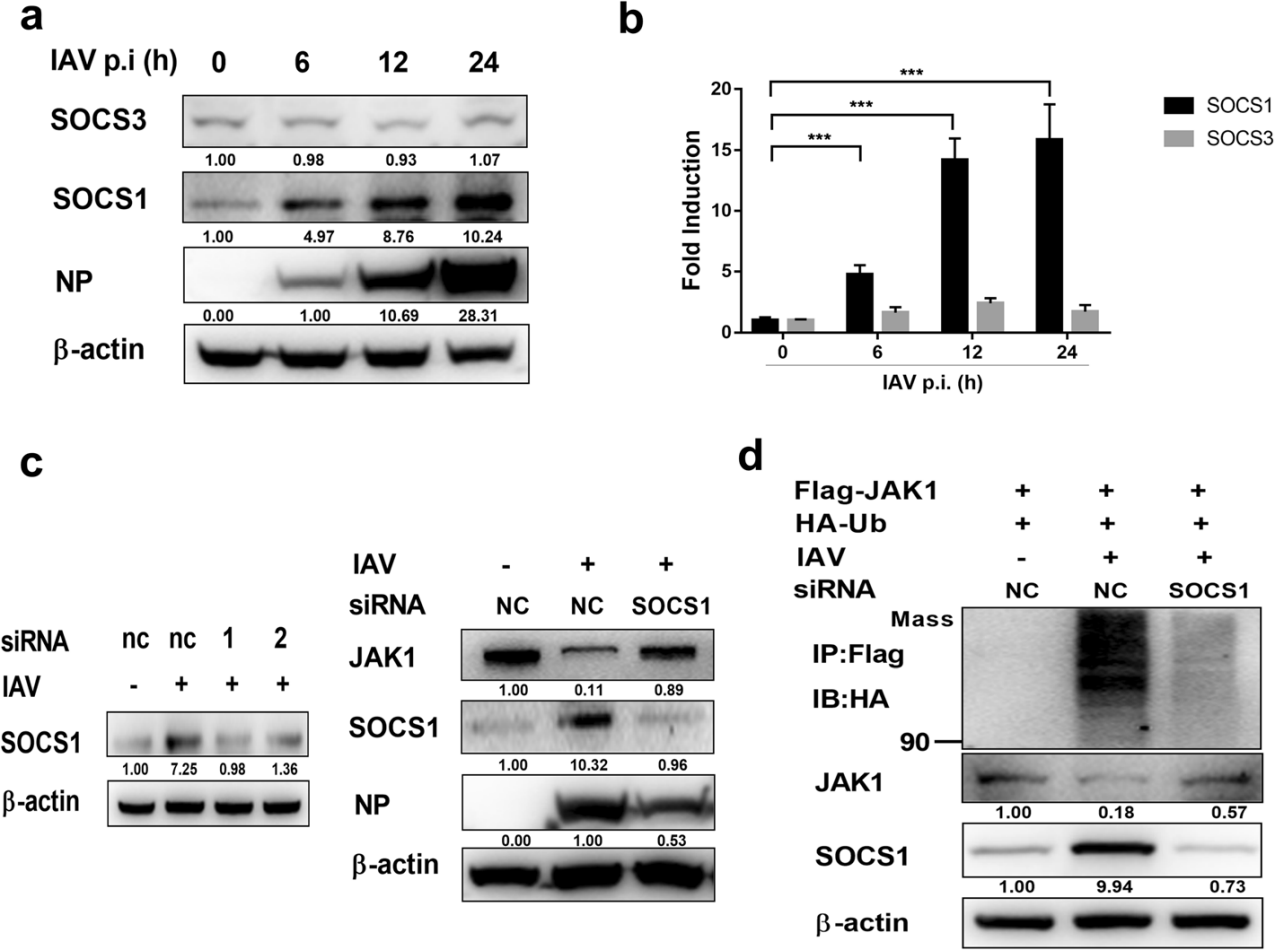
IAV infection induces the expression of SOCS1, resulting in JAK1 degradation. (**a**) 293 T cells were left uninfected or infected with IAV at an MOI of 1 for the indicated times. The protein levels of SOCS1, SOCS3, viral NP and β-actin were analyzed using Western blotting. (**b**) 293 T cells were left uninfected or infected with IAV at an MOI of 1 for the indicated times. The mRNA levels of SOCS1, SOCS3 were analyzed using real-time qPCR. The error bars represent the means plus standard deviations for three independent experiments. ***, P < .0001. (**c**) 293 T cells were transfected with siRNAs 1 and 2 targeting SOCS1 or NC (negative control) siRNA. Twenty-four hours later, cells were left uninfected or infected with IAV at an MOI of 1. The protein levels of SOCS1 and β-actin were analyzed by Western blotting at 24 hpi. 293 T cells were transfected with siRNAs 1 targeting SOCS1 or NC siRNA. Twenty-four hours later, cells were left uninfected or infected with IAV at an MOI of 1, and the protein level of SOCS1, JAK1, viral NP and β-actin were detected at 24 hpi. (**d**) 293 T cells were transfected with siRNA 1 targeting SOCS1 or NC siRNA as indicated. Six hours later, cells were transfected with Flag-tagged JAK1 and HA-tagged ubiquitin. Twenty-four hours post-transfection, cells were left uninfected or infected with IAV at an MOI of 1. At 18 h post infection, the cells were harvested and subjected to immunoprecipitation. The ubiquitination of immunoprecipitated Flag-JAK1 was analyzed using Western blotting using anti-HA tag antibody. The protein level of Flag-JAK1, SOCS1 and β-actin in the whole-cell lysates were also analyzed using Western blotting

Previous studies revealed that SOCS1 and SOCS3 target JAK1 for degradation [23, 24]. IAV infection upregulated SOCS1 and SOCS3, and SOCS1/3 antagonist peptide could protect mice against lethal influenza infection [25, 26]. Therefore, we investigated whether SOCS1 and SOCS3 are involved in the degradation of JAK1 during IAV infection. The protein level of SOCS1 was significantly increased during IAV infection, and the protein level of SOCS3 was not changed (Fig. 5a). IAV infection also upregulated the mRNA level of SOCS1 (Fig. 5b). To further verify the correlation of SOCS1 protein expression and JAK1 degradation, SOCS1 protein level in 293 T cells were knocked down using siRNA (Fig. 5c). In SOCS1 knockdown cells, the protein level of JAK1 was notably higher than that of control cells (Fig. 5c), indicating that SOCS1 directly mediated JAK1 degradation during IAV infection. Importantly, knocked-down of SOCS1 inhibited the expression of virus protein NP upon IAV infection (Fig. 5c). These results indicate that IAV infection induced SOCS1 is a negative regulator of cellular antiviral activity, and SOCS1 plays an important role in downregulating the protein level of JAK1. To determine if SOCS1 mediates JAK1 ubiquitination and degradation, 293 T cells were transfected with siRNA targeting SOCS1, then transfected with Flag-tagged JAK1 and HA-tagged ubiquitin, and then infected with IAV. SOCS1 knockdown attenuated the ubiquitination of JAK1 during IAV infection (Fig. 5d). Collectively, these results indicate that SOCS1 mediates the ubiquitination and degradation of JAK1 during IAV infection.

### Knock down of SOCS1 rescues IFNs response

**Fig. 6:**
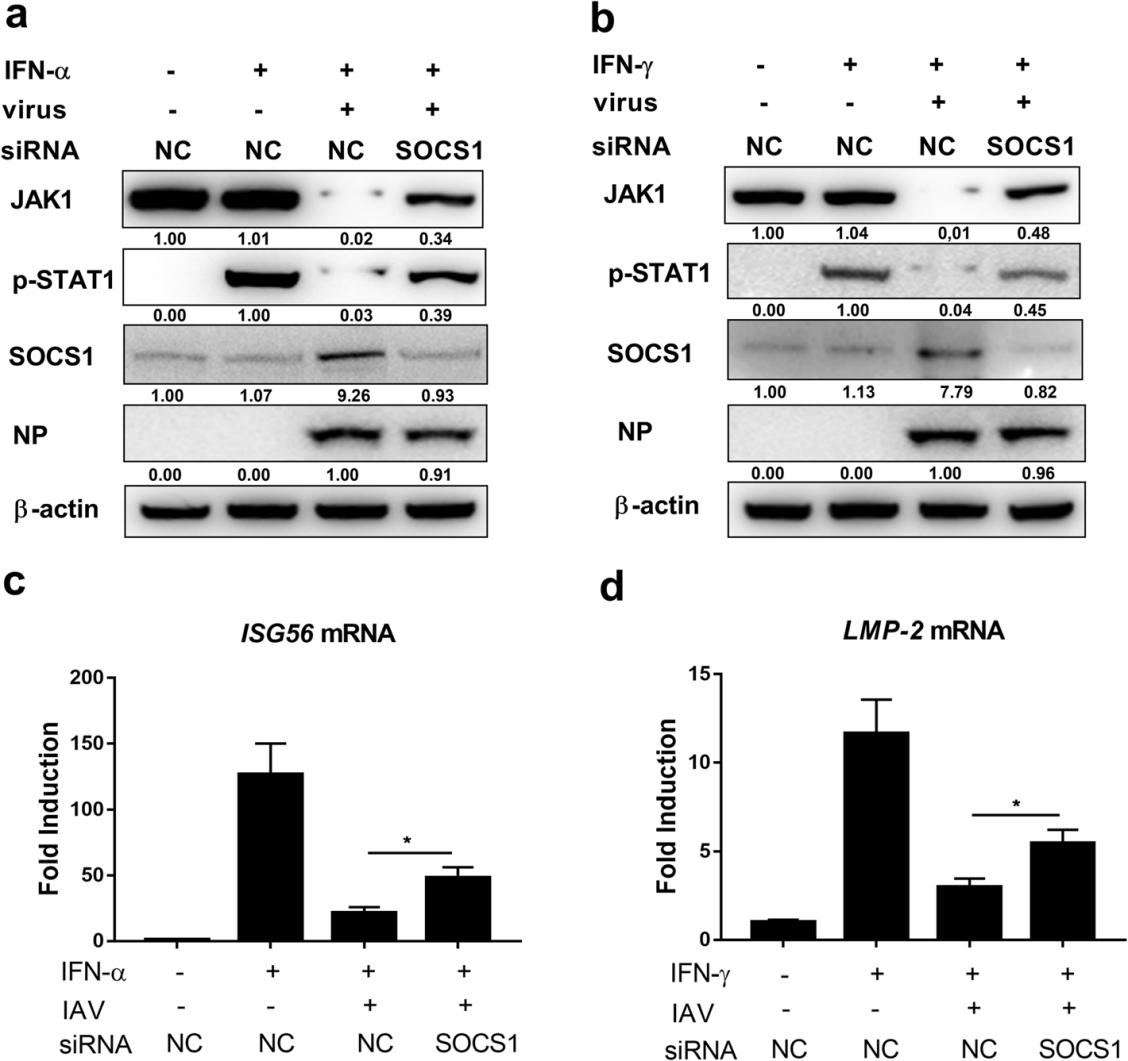
SOCS1 mediates the inhibition of IFNs response. (**a** and **b**) 293 T cells were transfected with siRNA 1 targeting SOCS1or NC siRNA. 24 h later, cells were left uninfected or infected with IAV at an MOI of 1. Twenty-four hours post infection, cells were left untreated (−) or treated with human IFN-α (1000 U/ml) (**a**) or IFN-γ (1000 U/ml) (**b**) for 1 h, and the protein level of JAK1, pSTAT1, SOCS1, viral NP and β-actin were analyzed using Western blotting. (c and d) 293 T cells were transfected with siRNA 1 targeting SOCS1or NC siRNA. Six hours later, 293 T cells were left uninfected or infected with IAV at an MOI of 1. At 24 hpi, cells were left untreated (−) or treated with human IFN-α (1000 U/ml) for 24 h or IFN-γ (1000 U/ml) for 6 h. The relative mRNA levels of ISG56 (c) or LMP-2 (**d)** were analyzed using real-time qPCR. The error bars represent the means plus standard deviations for three independent experiments. *, *p* ≤ 0.05

To further ascertain the importance of SOCS1 in inducing JAK1 degradation during IAV infection, SOCS1 were knocked down using siRNA before testing the cellular response to IFNs. SOCS1 knockdown rescued the protein level of JAK1 as well as the phosphorylation of STAT1 induced by type I and type II IFNs (Fig. 6a and b). To further investigate the association between SOCS1 and IFNs response inhibition, the expression level of ISGs were detected by real-time PCR; SOCS1 knockdown enhanced the expression of ISGs induced by IFNs (Fig. 6c and d), indicating that SOCS1 knock down can enhance the IFNs induced ISGs expression during IAV infection. Collectively, these results illustrate that SOCS1 expression induced by IAV infection can downregulate the protein level of JAK1, making cells less responsive to IFNs.

## Discussion

Zoonotic strains of influenza A virus remain important threats to global health, especially those strains that cause significant morbidity and mortality. Upon virus infection, interferon responses are induced in host cells to prevent viral replication, but IAV can evade cellular IFN response to propagate in host cells. Influenza A virus has evolved diverse strategies to inhibit the synthesis of IFNs, but how IAV evades the down-stream signaling pathway of IFNs is not well elucidated. In this study, we found that IAV infection induces JAK1 degradation in several cell types, including A549, 293 T and MDCK cell lines. Overexpression of JAK1 could partially restore the activation of STAT1 in response to IFN-α and IFN-γ, indicating that JAK1 degradation was responsible for the attenuated cellular IFN response during IAV infection.

JAK1 is a key regulator of interferon response and immune cell activation. Upon receptor ligation by IFNs, interferon receptors activate the catalytic activities of receptor associated Janus Kinase (JAK) family of tyrosine kinases to transduce signals [27]. Tyrosines in the cytoplasmic regions of the receptors are phosphorylated by activated JAKs, subsequently recruiting and phosphorylating STAT factors. Activated STATs translocate into the nucleus and then function as transcription factors, regulating the transcription program of ISGs. ISGs play an important role in cleaning the infection by directly inhibiting virus replication and regulating the antiviral immune functions. JAK1 can also mediate intracellular signaling from multiple cytokine receptors, such as IL-6 family cytokines, IL-10 family cytokines, IL-2, IL-4, IL-7, IL-9, IL-15, IL-21, and IL-27 [28]. Some of those cytokines are involved in the “cytokine storm” induced by IAV infection and play diverse roles in influenza immune responses. Further studies should be done to evaluate the importance of JAK1 degradation induced by IAV infection.

Many viruses attenuate cellular responses to IFNs by targeting proteins of IFNs signaling pathway for degradation. On the receptor level, interferon receptors degradation are induced during IAV, HSV (Herpes simplex virus), HCV (Hepatitis C virus), and VSV (Vesicular stomatitis virus) infection [29]. IFNs activated STAT1 is a target for ubiquitylation and degradation during multiple viruses infection [30]. Human Metapneumovirus (hMPV) enhances the proteasomal degradation of JAK1 protein in A549 cells, and then inhibits IFN-β stimulated antiviral and immunoregulatory activity [15]. Foot-and-mouth disease virus (FMDV) degrades JAK1 via lysosomal pathway to inhibit IFN-γ signaling transduction pathway [14]. Zika virus (ZIKV) suppresses JAK/STAT signaling by targeting JAK1 for proteasomal degradation, impairing interferon mediated antiviral response [16]. Human cytomegalovirus (HCMV) induces proteasome-dependent degradation of Jak1, inhibiting IFN-α signal transduction pathway [17]. SOCS family proteins mainly induced by IFNs are the most well-described negative regulation factors of JAK/STAT signaling, and can be a negative feedback on the IFNs response during virus infection [31, 32]. There are eight numbers in the SOCS family, and SOCS1 is the most extensively studied inhibitor of JAK/STAT pathway. SOCS1 can directly bind to IFNAR1 and IFNGR1 and then inhibit IFNs mediated activities [33, 34]. SOCS1 can also directly interact with JAKs, resulting in inhibition of JAKs activity [35]. Several studies showed that SOCS1 targeted JAK1 to the proteasome for degradation [36, 37]. Although influenza A virus was previously reported to reduce the protein level of JAK1 [38], how the virus might inhibit JAK1 and the influence of JAK1 downregulation during IAV infection were not assessed. IAV infection could induce the expression of SOCS genes, the kinds and multiples of SOCS genes upregulation were different due to the variety of virus strains and cells [25, 39–42]. Our data showed that IAV infection induced expression of SOCS1, not SOCS3, and SOCS1 mediated the degradation of JAK1. During IAV infection, overexpression of JAK1 or knockdown of SOCS1 can partially restore the cellular interferon response, activating the phosphorylation of STAT1, enhancing the transcription of antiviral ISGs. Previous studies showed that IAV induced SOCS genes expression dependent on IFNs or other cytokines such as IL-17A [25, 43], and some other studies showed that IAV upregulated SOCS genes dependent on IAV itself or the viral 5′ triphosphate RNA, especially at the early stage of IAV infection, [41, 44]. In our study, SOCS1 expression was significantly upregulated as earlier as 6 h post IAV infection, it was more likely that IAV directly induced SOCS1 expression and SOCS1 mediated JAK1 degradation, further experiments need to be done to clarify the mechanism.

Protein ubiquitylation is crucial for regulating IFNs response via inducing protein degradation. Protein ubiquitylation involves conjugation of ubiquitin to the substrate proteins. SOCS box proteins can function as adaptors of the E3 ubiquitin ligase complex through interacting with Elongin B/C complex, and then target the substrate proteins for ubiquitination and degradation [45]. Recently, the interaction of SOCS1, JAK1, and Elongin B/C was elucidated, demonstrating regulation of JAK1 by SOCS1 [35]. In this study, we found that IAV induced SOCS1 to mediate the ubiquitination and degradation of JAK1, resulting in inhibition of IFNs activity. A previous study showed that E3 ubiquitin ligase RNF125 (ring finger protein 125) bound to JAK1 and promoted its ubiquitination and degradation [46]. Nedd4 family E3 ubiquitin ligases can mediate the degradation of JAK1 and restrict cytokine signaling to limit T cells expansion [47]. It will be interesting to further investigate whether RNF125, Nedd4 family E3 ubiquitin ligases, and other E3 ubiquitin ligases, participate in SOCS1 mediated degradation of JAK1 during IAV infection.

## Conclusions

Taken together, our results revealed that IAV infection induced SOCS1 to attenuate cellular responses to IFNs by targeting JAK1 for degradation. Investigating the JAK1 elimination induced by IAV could help us better understand the pathogenesis of IAV and might provide new therapeutic targets for treating influenza.

## Data Availability

All data generated or analyzed during this study are included in this published article.

## Abbreviations

IAV: Influenza A virus
IFN: Interferon;
JAK1: Janus kinase 1
STAT1: Signal transducer and activator of transcription 1
SOCS: suppressor of cytokine signaling
ISG: Interferon stimulated gene
PRRs: pathogen recognition receptors
CHX: Cycloheximide
MG132: Carbobenzoxy-L-leucyl-L-leucyl-L-leucinal
NH4Cl: Ammonium chloride
h: hour
MOI: multiplicity of infection
siRNA: small interfering RNA
Ub: ubiquitin
HSV: Herpes simplex virus
HCV: Hepatitis C virus
VSV: Vesicular stomatitis virus
hMPV: human Metapneumovirus
FMDV: Foot-and-mouth disease virus
ZIKV: Zika virus
HCMV: Human cytomegalovirus
RNF125: ring finger protein 125.
